# Epigallocatechin-3-gallate and Epigallocatechin-3-*O*-(3-*O*-methyl)-gallate Enhance the Bonding Stability of an Etch-and-Rinse Adhesive to Dentin

**DOI:** 10.3390/ma10020183

**Published:** 2017-02-15

**Authors:** Hao-Han Yu, Ling Zhang, Fan Yu, Fang Li, Zheng-Ya Liu, Ji-Hua Chen

**Affiliations:** State Key Laboratory of Military Stomatology & National Clinical Research Center for Oral Diseases & Shaanxi Key Laboratory of Stomatology, Department of Prosthodontics, School of Stomatology, Fourth Military Medical University, 145 West Changle Road, Xi’an 710032, China; yhh.king@foxmail.com (H.-H.Y.); zhang.ling1978@gmail.com (L.Z.); 81811@fmmu.edu.cn (F.Y.); lifangfoues@aliyun.com (F.L.); luna6739@foxmail.com (Z.-Y.L.)

**Keywords:** antibacterial effect, dental adhesive, epigallocatechin-3-gallate, epigallocatechin-3-*O*-(3-*O*-methyl)-gallate, microtensile bond strength, thermocycling

## Abstract

This study evaluated epigallocatechin-3-gallate (EGCG) and epigallocatechin-3-*O*-(3-*O*-methyl)-gallate (EGCG-3Me) modified etch-and-rinse adhesives (Single Bond 2, SB 2) for their antibacterial effect and bonding stability to dentin. EGCG-3Me was isolated and purified with column chromatography and preparative high performance liquid chromatography. EGCG and EGCG-3Me were incorporated separately into the adhesive SB 2 at concentrations of 200, 400, and 600 µg/mL. The effect of cured adhesives on the growth of *Streptococcus mutans* (*S. mutans*) was determined with scanning electron microscopy and confocal laser scanning microscopy; the biofilm of bacteria was further quantified via optical density 600 values. The inhibition of EGCG and EGCG-3Me on dentin-originated collagen proteases activities was evaluated with a proteases fluorometric assay kit. The degree of conversion (DC) of the adhesives was tested with micro-Raman spectrum. The immediate and post-thermocycling (5000 cycles) bond strength was assessed through Microtensile Bond Strength (MTBS) test. Cured EGCG/EGCG-3Me modified adhesives inhibit the growth of *S. mutans* in a concentration-dependent manner. The immediate MTBS of SB 2 was not compromised by EGCG/EGCG-3Me modification. EGCG/EGCG-3Me modified adhesive had higher MTBS than SB 2 after thermocycling, showing no correlation with concentration. The DC of the adhesive system was affected depending on the concentration of EGCG/EGCG-3Me and the depth of the hybrid layer. EGCG/EGCG-3Me modified adhesives could inhibit *S. mutans* adhesion to dentin–resin interface, and maintain the bonding stability. The adhesive modified with 400 µg/mL EGCG-3Me showed antibacterial effect and enhanced bonding stability without affect the DC of adhesive.

## 1. Introduction

Dental resinous composites are valued because of their excellent esthetics, direct-filling capabilities and load-bearing properties [1,2,3]. A dental resinous composite restores the appearance and function of teeth with extensive loss of the tooth structure by bonding to the tooth structure via a resinous adhesive [4]. The overall long-term performance of dental resinous restorations is dependent on the bonding interface formed between the restoration and the dentin, especially the dentin–adhesive interface [5]. However, nearly one-half of all restorations fail within 10 years, and replacing them accounts for 50%–70% of all restorations [6,7], which causes economic losses and waste of medical resources [6]. Secondary caries and dentin-originated collagen proteases -leading degradation of dentin–adhesive interfaces are the two major reasons that impair the longevities of dental resinous restorations [8,9].

Secondary caries is a main cause of a resinous restoration failure in caries-like patients [10] and may occur for various reasons. Resinous restorative materials have no antibacterial function, and even accumulate more biofilms/dental plaque in vivo when compared to silver amalgam or glass ionomer cements [11,12,13]. Cariogenic bacteria in biofilms produce acids that can partly demineralize dentin at the restoration margins and promote the adhesion of bacteria [14,15]. In addition, the inevitable polymerization shrinkage of resinous restoration materials generates stress within the resin and may lead to marginal gap formation and microleakages [16], thereby providing bacteria with an invading pathway and causing secondary caries [17]. Furthermore, with the popularization of a more conservative approach of caries removal [18], more affected tissue is saved during the surgical procedure and residual bacteria possibly harbors [19]. These remaining bacteria would begin to proliferate during the bonding operation or during the process of aging [20]. Thus, resinous restorations may be destroyed starting from the bottom. To inhibit the colonization of bacteria from the oral cavity or the prepared carious cavity, an adhesive system with antibacterial function is needed [20]. Incorporating antibacterial agents or antibacterial monomers in an adhesive effectively inhibits bacterial colonization and growth [10,21]. Chemical synthetics such as chlorhexidine (CHX), methacryloxyethyl cetyl dimethyl ammonium chloride (DMAE-CB), 12-methacryloyloxydodecyl-pyridinium bromide (MDPB) and quaternary ammonium dimethacrylate (QADM) have been used [10,21,22,23,24]. These efforts have proven that developing adhesives with antibacterial effects is a promising approach for preventing secondary caries.

The bonding of dentin to a resinous restoration depends on the penetration of an adhesive into the demineralized intertubular dentin, creating a complete and dense hybrid layer (HL) [5]. However, the bonding strength and the integrity of the HL are proved to decrease over time. The so-called “degradation of the hybrid layer” is the main limitation to the stability of dentin–adhesive interfaces [6]. Several factors are responsible for the degradation of the HL, such as the hydrolysis of the resin and the enzymatic degradation of exposed collagen [6]. Several enzymes, such as Cysteine Cathepsins (CTs) and Endogenous matrix metalloproteinase (MMP) [25,26,27], have been proved to take responsibility for the collagen degradation in the HL. Using MMP inhibitors has been reported to be an effective strategy to inhibit the degradation of exposed collagen and keep bonding stability [26,27,28,29]. Anti-MMP materials such as CHX, MDPB and dimethylamino dodecyl methacrylate have been incorporated into adhesives to inhibit MMP activity and thereby obtain a more stabilized and longer-lasting dentin–resin interface [28,29].

More than 50% of green tea contains EGCG, the main polyphenol component [30]. It is responsible for most potential health benefits of green tea, such as antioxidant, antidiabetic, anti-inflammatory, antimicrobial, protease inhibition effects, and cancer prevention [30,31,32,33]. EGCG has been proved to be promising in preventing dental erosion and maintaining the bonding stability of both etch-and-rinse system and self-etch system [32,34,35,36]. Kato et al. [32] found that gels containing EGCG can prevent dental erosion and Hongye et al. [35] proved that pretreatment with 0.02% EGCG/ethanol solutions can effectively improve immediate bond strength and bond stability of etch-and-rinse adhesives on dentin. A previous study also demonstrated that 200 µg/mL of an EGCG-modified adhesive could maintain the stability of the resin–dentin interface after six months of aging in distilled water [34]. There is no direct evidence to prove that EGCG can inhibit MMP in dentin; however, researchers attribute these effects to the inhibition of MMP by EGCG [34,35]. However, EGCG is unstable under alkaline or neutral conditions, which limits its usage [37]. To improve the stability and bioavailability of EGCG and endowed EGCG with new biological functions [37], researchers have tried to introduce new function groups to EGCG by chemical synthesis, such as acetylated EGCG, methylated EGCG, and glycosylated EGCG. Epigallocatechin-3-*O*-(3-*O*-methyl) gallate (EGCG-3Me), an *O*-methylated derivative of EGCG results from partial methylation of the hydroxyl group at the 3″ position in the galloyl moieties, shows better stability, bioavailability and absorption in blood than EGCG, also better antiallergy/anticytotoxic and antimycobacterium tuberculosis effects, as well as antiangiotensin I-converting enzyme activity [38,39,40]. We speculated that EGCG-3Me might perform better than EGCG in maintaining the stability of the dentin–adhesive interface. However, there has been no previous study about this topic.

In the present study, the dentin adhesive SB 2 was modified with EGCG and with EGCG-3Me to evaluate their antibacterial effects and their bonding stability to dentin spontaneously. Thus, the null hypotheses tested were: (1) the EGCG/EGCG-3Me modified adhesives would have no antibacterial effect; (2) the bonding stability of the adhesive would not be affected by EGCG/EGCG-3Me modification; and (3) there would be no difference in the antibacterial effect and dentin–adhesive interface stability between the two modified adhesives.

## 2. Materials and Methods

### 2.1. Preparation of Adhesives

**Figure 1:**
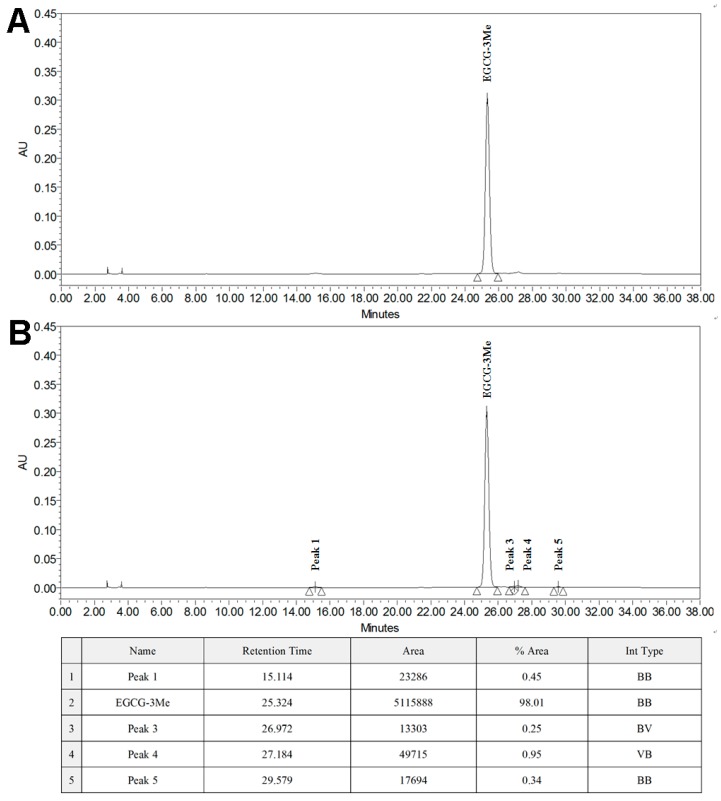
The HPLC chromatograms of: the EGCG-3Me standard (**A**) and the isolated compound containing EGCG-3Me (**B**). EGCG, epigallocatechin-3-gallate; EGCG-3Me, epigallocatechin-3-*O*-(3-*O*-methyl)-gallate; HPLC, high-performance liquid chromatography.

Adper Single Bond 2 (SB 2) (3M ESPE, St. Paul, MN, USA), a commercially available etch-and-rinse adhesive system with ethanol as the solvent, was used as the control. The composition and application procedures of SB 2 are presented in Table 1. EGCG and EGCG-3Me were used as the specific functional additives to SB 2. EGCG is a commercially available tea extract from Sigma-Aldrich (E4143, Sigma-Aldrich Corp, St. Louis, MO, USA) and EGCG-3Me was isolated using column chromatography and preparative high-performance liquid chromatography (HPLC) method as described previously [41]. Pulverized China oolong tea (200 g) was wetted with 200 mL distilled water and further extracted with ethylacetate (TIANLI Chemical Reagents Ltd., Tianjin, China). The concentrated crude extract was separated on an HP-20 column by eluting with gradient aqueous alcohol. An eluate of 80% ethanol was gathered and concentrated. Various concentrations were then subjected to preparative HPLC to isolate the compound that contained EGCG-3Me. The chromatogram of the compound was compared with that of the standard EGCG-3Me (EMMX Biotechnology LLC., Lake Forest, CA, USA). They had the same peak appearance time and EGCG-3Me accounted for 98.01% of the total quality of the compound (Figure 1).

EGCG and EGCG-3Me were dissolved in absolute ethyl alcohol (Kermel Chemical Reagent Co., Inc., Tianjin, China) at the concentrations of 10 mg/mL, 20 mg/mL, and 30 mg/mL to prepare the EGCG/EGCG-3Me ethanol solutions. The obtained solutions at the different concentrations were mixed with the adhesive SB 2 at a volume ratio of 2% in a dark room to get the final concentrations of EGCG /EGCG-3Me at 200 μg/mL, 400 μg/mL, and 600 μg/mL, based on the method used in previous studies [34]. Thus, the following seven adhesives were formulated:

(1) Adper SB 2 as the control (SB 2);
(2) SB 2 + 200 μg/mL EGCG (EGCG 200);
(3) SB 2 + 400 μg/mL EGCG (EGCG 400);
(4) SB 2 + 600 μg/mL EGCG (EGCG 600);
(5) SB 2 + 200 μg/mL EGCG-3Me (EGCG-3Me 200);
(6) SB 2 + 400 μg/mL EGCG-3Me (EGCG-3Me 400);
(7) SB 2 + 600 μg/mL EGCG-3Me (EGCG-3Me 600).

### 2.2. Antibacterial Test

#### 2.2.1. Specimens Preparation

The cover of a sterile 96-well plate (Corning/Costar, New York, NY, USA) was used for specimen preparation [42]. A total volume of 30 μL of the adhesives was added to the round indentions on the cover with a micropipette (Eppendorf, Hamburg, Germany). All visible air bubbles were carefully removed using a hypodermic needle (Weigao Group Medical Polymer Co., Ltd., Weihai, China). The adhesive was photopolymerized for 10 seconds (s) by a light-emitting diode (LED) curing light (Elipar S10; 3M ESPE) with a polyester strip covering to obtain a specimen disk of 8 mm in diameter and 0.5 mm thick. The power density of the light was maintained at 1200 mW/cm^2^. All specimens were immersed in distilled water at 37 °C and agitated constantly with a magnetic stirrer (CJJ 78-1; Shuai Deng Instrument Co., Ltd., Shanghai, China) for 1 hour (h) to remove unpolymerized monomers [43]. The obtained disks were dried at room temperature and then were sterilized using ultraviolet light for 1 h [34].

#### 2.2.2. Bacteria Culture and Inoculation

*Streptococcus mutans* UA 159 (*S. mutans*, ATCC #700610) used in this study was approved by the Fourth Military Medical University (Xi’an, China). *S. mutans* was cultured overnight at 37 °C in brain heart infusion broth (BHI, Qingdao Hope Bio-Technology Co., Ltd., Qingdao, China) in an anaerobic system (Don Whitley Scientific, H85, West Yorkshire, UK). The bacterial suspension was adjusted to an optical density of 0.5 at 600 nm (OD 600) for further usage [44].

Fifteen disks from each group were placed in the wells of 24-well plates (Corning/Costar) with 2 mL of BHI supplemented with sucrose (Tianjin Fuchen Chemical Reagents Factory, Tianjin, China) at a mass-volume concentration of 0.2%. The bacterial suspension prepared beforehand was diluted to 1:100, and 10 µL of the suspension was then inoculated into each well. After incubation for 24 h, the biofilm on the disks was used in the following experiments [44].

#### 2.2.3. OD 600 Evaluation

Five disks were selected randomly from each group. The adherent bacteria were detached by vibrating them with a sonifier cell disruptor (CLN-400S; Kelaien Experimental Instrument Co. Ltd., Tianjin, China) in 1 mL of distilled water. The OD 600 value of the bacterial suspension was measured with a microplate spectrophotometer (PowerWave 340; BioTek Instruments, Winooski, VT, USA) [34].

#### 2.2.4. Scanning Electron Microscopy Observation

Five disks from each group were used to observe the bacterial biofilm via scanning electron microscopy (SEM). The disks were gently rinsed with distilled water and fixed in 2.5% glutaraldehyde (Tianjin Fuchen Chemical Reagents Factory, Tianjin, China) at 4 °C overnight. Specimens were then dehydrated in ascending ethanol series, followed by desiccation with a lyophilizer (Hitachi E-2030, Tokyo, Japan). After being mounted on aluminum stubs and sputter-coated (Hitachi E-1045; Hitachi, Tokyo, Japan), the specimens were observed with SEM (FE-SEM 4800; Hitachi, Tokyo, Japan).

#### 2.2.5. Confocal Laser Scanning Microscope Observation

Another five disks from each group were rinsed in distilled water for 1 min to remove nonadherent bacteria. The biofilms were stained with a live/dead bacterial kit (LIVE/DEAD BacLight Bacterial Viability Kit L7012; Molecular Probes, Inc., Eugene, OR, USA). Live bacteria were stained with SYTO 9, which produces a green fluorescence, and bacteria with compromised membranes were stained with propidium iodide, which produces a red fluorescence. In accordance with the manufacturer’s instructions, the bacterial biofilm on each disk was stained away from light for 15 min. Confocal laser scanning microscope (CLSM) (FluoView FV1000; Olympus, Tokyo, Japan) was used to view the fluorescence of the stained bacteria.

### 2.3. Microtensile Bond Strength Test

Seventy noncaries human third molars were used for the microtensile bond strength (MTBS) test. The molars were freshly extracted (within 1 week) and stored in 0.9% physiological saline containing 0.002% sodium azide (Micxy Reagent, Chengdu, China) at 4 °C before use. All teeth were collected after obtaining the patients’ informed consent and the protocol employed was approved by the Ethic Committee for Human Studies of the Fourth Military Medical University (Xi’an China). All experiments were conducted in accordance with the approved guidelines and regulations.

Using a water-cooled low-speed cutting saw (SYJ-150; MTI Corp., Shenyang, China), the roots and the occlusal one-third of the tooth crown were removed to expose a flat dentin surface. The dentine surface was polished with 600-grit silicon carbide (SiC) paper to create a standardized smear layer and then was treated with seven experimental adhesives using the etch-and-rinse bonding technique. In brief, the dentin surfaces were acid-etched by 37% phosphoric acid gel (DenFil Etchant-37; Vericom Co., Ltd., Gyeonggi-do, Korea) for 15 s and then rinsed with distilled water for 10 s. After blot drying with the wet bonding technique, two layers of adhesives from each group were applied with microbrushes on the dentin surface. After being air-dried with a gentle air stream for 10 s, the disks were light cured via an LED curing light. Three millimeters of Z250 resin composite (3M ESPE) were used to rebuild the bonding area. After being stored in deionized water at 37 °C for 24 h, each tooth was vertically sectioned into slabs with a thickness of 0.9 mm and further sectioned into 0.9 mm × 0.9 mm composite-dentine sticks with a water-cooled low-speed cutting saw. Sticks with enamel were excluded. Sticks from one tooth were randomly separated into two groups: the first group underwent immediate testing; and the second group underwent testing via thermocycling for 5000 cycles.

Eighty bonded sticks from each group were attached to the testing apparatus with cyanoacrylate glue and were stressed to failure under uniaxial tension in a computer-controlled testing machine (EZ-Test; Shimadzu, Tokyo, Japan) at the crosshead speed of 1 mm/min. The exact areas of each stick were measured with a digital caliper (605-04; Harbin Measuring and Cutting Tool Group Co., Ltd., Harbin, China). The MTBS was calculated by the equation MTBS = F/S, in which “F” is the maximum load at failure and “S” is the cross-sectional area of the fracture interface.

The sample size of MTBS test was determined by preliminary power analysis, which revealed that having at least nine specimens per final experimental group would assure a power of 90% for finding the statistical significance of two factors and the significance of their interactions in a two-way (2 × 7) analysis of variance (ANOVA) design, at a 0.001 value of type 1 errors. A difference of 7 MPa for MTBS was assumed as relevant based on the findings of our pilot study. The calculations were handled by PASS Power Analysis software, version 11.0 (NCSS; Kaysville, UT, USA).

### 2.4. Micro-Raman Analysis for the Degrees of Conversion Measurements

Twenty-eight dentin slices from human third molars were obtained midcoronally with a water-cooled low-speed diamond saw. They were used for the degree of conversion (DC) measurement.

For each group, 5 µL of adhesive was applied to the slices. Before curing, one specimen of each group was evaluated using a micro-Raman spectrometer (HR800; HORIBA Jobin Yvon, Paris, France) with a laser wavelength of 633 nm and exposure time of 60 s to acquire the reference peak($r_{1608\;{\text{cm}}^{-1}}$) and reaction peak($r_{1640\;{\text{cm}}^{-1}}$) of the adhesives. Resinous composites were adhered to the surface of the other three specimens as described for the MTBS test, and then sectioned into slices along the direction vertical to the bonding interface. The slice in the middle was used for the DC measurement. The exposed bonding interfaces were ground flat using silicon carbide paper up to 4000-grit and then polished with diamond paste to 0.25 µm. The polished surfaces were analyzed to acquire the reference peak ($R_{1608\;{\text{cm}}^{-1}}$) and reaction peak ($R_{1640\;{\text{cm}}^{-1}}$) of the cured adhesives by collecting the spectrum in three line scans at three random positions, starting from the resin composite and ending in the sound mineralized dentin. A computer-controlled motorized x-y-z stage was used to conduct the line scans vertical to the dentin–adhesive interface. To evaluate the DC of different depths in the HL, three points along the HL were selected and identified: (1) the adhesive-composite interface; (2) the middle of the HL; and (3) the bottom of the HL that adjoins the dentin. The mean values of each point were calculated as the final values.

The DC was calculated with the following formula [45]: DC (%) = [1 − ($R_{1640\;{\text{cm}}^{-1}}$/$R_{1608\;{\text{cm}}^{-1}}$)/($r_{1640\;{\text{cm}}^{-1}}$/$r_{1608\;{\text{cm}}^{-1}}$)] × 100(%), in which R is the Raman intensity of aliphatic and aromatic band after the adhesives have been cured and r is the Raman intensity of aliphatic and aromatic band before the adhesives are cured.

### 2.5. Inhibition of Dentin-Originated Collagen Proteases Activities

EnzChek Collagenase/Gelatinase kits (E12055; Molecular Probes, Eugene, OR, USA) were used to determine the inhibition of dentin-originated collagen proteases with DQ™ gelatin as the substrate.

#### 2.5.1. Preparation of Dentin Powder

Dentin powder was acquired by the liquid nitrogen grinding method [46]. The dentin powder was completely demineralized in 10 wt % phosphoric acid for 8 h at 25 °C with magnetic stirring [47]. The demineralized dentin powder was isolated via centrifugation (30 min at 3000 rpm), and then suspended and rinsed for three times in distilled water (20 mL) before a final centrifugation to remove the residual phosphoric acid. After drying in an oven at 37 °C for 8 h, the dentin powder was weighed and separated into eight groups (400 mg for each group). Each group had five replicates (80 mg for one replicate).

#### 2.5.2. Preparation of the Experimental Solutions

Experimental solutions were prepared based on the concentration of EGCG/EGCG3-Me. EGCG and EGCG3-Me were dissolved in deionized water at the concentrations of 200 µg/mL, 400 µg/mL, and 600 µg/mL. A general inhibitor of metalloproteinases, 1,10-phenanthroline, was served as positive control (provided by the assay kits) and distilled water was served as the negative control. Therefore, eight solutions were formulated:

(1) Distilled water (negative control);
(2) 1,10-phenanthroline (positive control);
(3) 200 μg/mL EGCG (EGCG 200);
(4) 400 μg/mL EGCG (EGCG 400);
(5) 600 μg/mL EGCG (EGCG 600);
(6) 200 μg/mL EGCG-3Me (EGCG-3Me 200);
(7) 400 μg/mL EGCG-3Me (EGCG-3Me 400);
(8) 600 μg/mL EGCG-3Me (EGCG-3Me 600).

#### 2.5.3. Treatment of the Dentin Powder

Dentin powder was treated with 5 mL experiment solution for 60 s and then rinsed with distilled water, further centrifuged for five times (30 min at 3000 rpm). After dried in an oven at 37 °C for 8 h, the treated dentin powder was added into the holes of a black 96-well plate, five for each group. In accordance with the assay kit manufacturer’s instruction, 180 µL of the reaction buffer and 20 µL fluorescent substrates were added to the hole of a black 96-well plate. The assay was performed in five replicates for each group. A blank control containing no dentin powder was set in each group to eliminate the background fluorescent intensity. The fluorescent intensity was measured programmatically at 1 h, 4 h, 8 h, 12 h, 24 h and 48 h with the Varioskan Flash system (Thermo Scientific, Waltham, MA, USA). The reaction temperature was maintained at 37 °C. The excitation/emission wavelengths were 495/515 nm. The background fluorescent intensity of each group was determined separately and subtracted from the wells at each time point. The inhibition of collagen proteases activities by the positive control and experimental groups was expressed as the percentage of the fluorescence of the negative control [48].

### 2.6. Statistical Analysis

The data of OD 600, MTBS, DC evaluation, and percent inhibition of dentin-originated collagen proteases activities were first verified by the Kolmogorov–Smirnov test for their normal distribution and by the Levene test for the homogeneity of variances. The results of OD 600 were then analyzed using one-way ANOVA with OD 600 as the dependent variable and the adhesive as the fixed factor. The results of MTBS test, DC evaluation, and percent inhibition of dentin-originated collagen proteases were analyzed using two-way ANOVA. For MTBS, the adhesive and time were the fixed factors. For the DC evaluation, the adhesive and position of the HL were the fixed factors. For the inhibition of dentin-originated collagen proteases, the treatment solution and time were the fixed factors. The Tukey test was used for post hoc comparisons. In all tests, the level of significance was analyzed with SPSS software, version 17.0 (SPSS, Chicago, IL, USA).

## 3. Results

### 3.1. Isolation of EGCG-3Me

Figure 1 shows the HPLC chromatograms of the EGCG-3Me standard (Figure 1A) and a compound containing EGCG-3Me (Figure 1B). The main substance in the compound had the same peak with the EGCG-3Me standard. The table in Figure 1 shows that the isolated compound contained more than 98% of EGCG-3Me.

### 3.2. Antibacterial Test

The mean OD 600 values and standard deviations are displayed in Table 2. SB 2 had the highest OD 600 value than other group (*p* < *0*.05) except for EGCG 200 (*p* = 0.089). The OD 600 value was lower for EGCG-3Me than for EGCG at the same concentrations (*p* < 0.05). The OD 600 value was lowest for EGCG-3Me 600 (*p* < 0.05).

**Figure 2:**
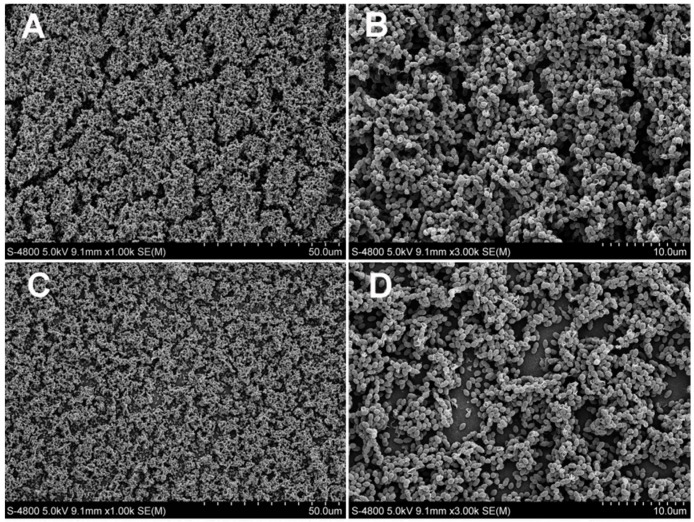

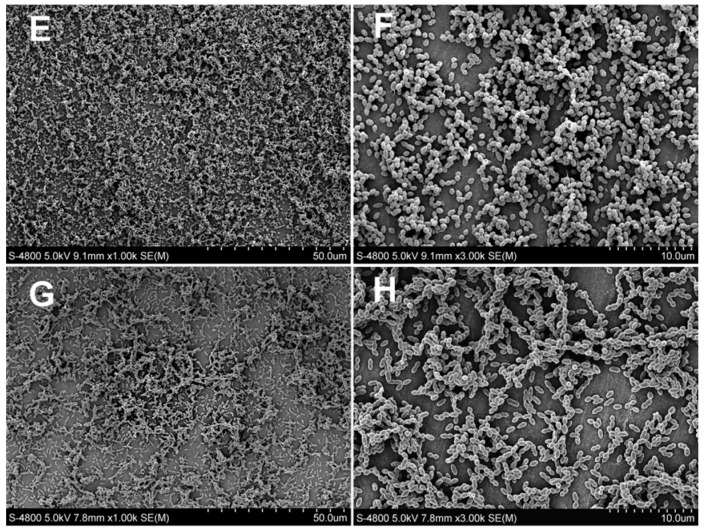
The scanning electron micrographs (magnification 1000× and 3000×) of *Streptococcus mutans* biofilms on the specimens in the control group and in the EGCG groups: SB 2 (**A**,**B**); EGCG 200 (**C**,**D**); EGCG 400 (**E**,**F**); and EGCG 600 (G,**H**). Dense biofilms have formed on the surface of the SB 2 specimens. EGCG 200, 400, and 600, epigallocatechin-3-gallate at 200 µg/mL, 400 µg/mL, and 600 µg/mL, respectively; EGCG-3Me 200, 400, and 600, epigallocatechin-3-*O*-(3-*O*-methyl)-gallate at 200 µg/mL, 400 µg/mL, and 600 µg/mL, respectively; SB 2, Single Bond 2 (3M ESPE, St. Paul. MN, USA).

**Figure 3:**
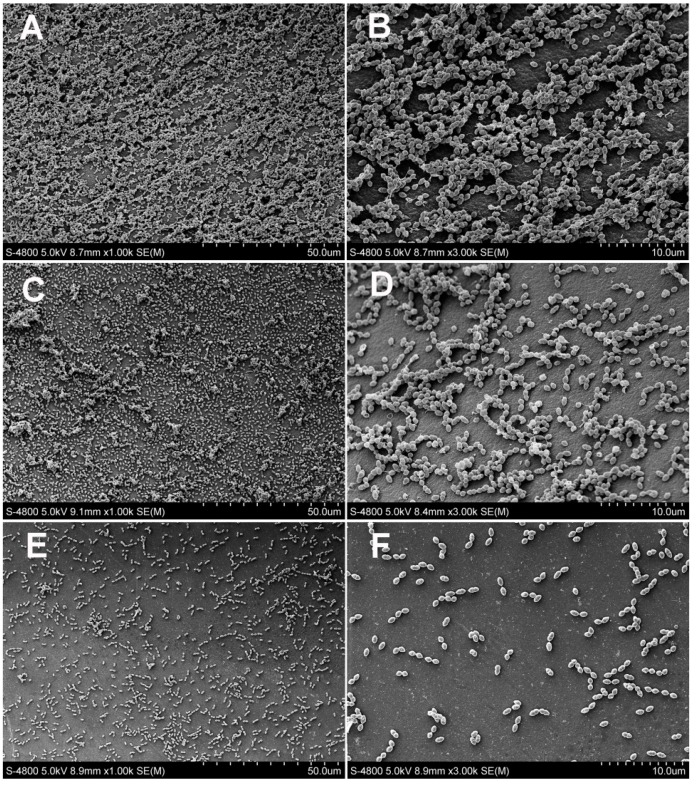
Scanning electron micrographs (magnification, 1000× and 3000×) of *Streptococcus mutans* biofilms on the specimens of the EGCG-3Me groups: EGCG-3Me 200 (**A**,**B**); EGCG-3Me 400 (**C**,**D**); and EGCG-3Me 600 (**E**,**F**). EGCG 200, 400, and 600, epigallocatechin-3-gallate at 200 µg/mL, 400 µg/mL, and 600 µg/mL, respectively; EGCG-3Me 200, 400, and 600, epigallocatechin-3-*O*-(3-*O*-methyl)-gallate at 200 µg/mL, 400 µg/mL, and 600 µg/mL, respectively; SB 2, Single Bond 2 (3M ESPE, St. Paul. MN, USA).

The SEM images (magnification, 1000× and 3000×) are presented in Figure 2 (SB 2 and EGCG groups) and Figure 3 (EGCG-3Me groups). In the control group (Figure 2A,B), dense biofilms were attached to the surface of specimen. Few bacteria were physiologically dead. EGCG and EGCG-3Me inhibited the biofilm formation and this inhibition was concentration-dependent. The amount of dead bacteria residue increased at higher concentrations of EGCG/EGCG-3Me. At the same concentrations, the biofilm was scantier with EGCG-3Me than with EGCG. Only a few bacteria were adherent to the surface of EGCG-3Me 600 group (Figure 3E,F).

**Figure 4:**
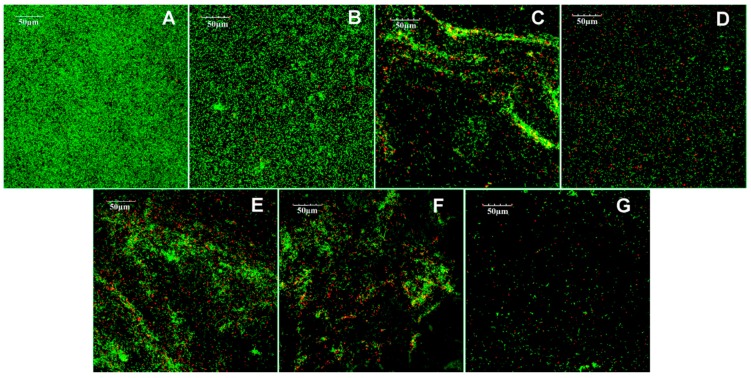
Confocal laser scanning microscope images of the *Streptococcus mutans* biofilms on the specimens: SB 2 (**A**); EGCG 200 (**B**); EGCG 400 (**C**); EGCG 600 (**D**); EGCG-3Me 200 (**E**); EGCG-3Me 400 (**F**); and EGCG-3Me 600 (**G**). Green fluorescence indicates the live bacteria and red fluorescence indicates the dead bacteria. EGCG 200, 400, and 600, epigallocatechin-3-gallate at 200 µg/mL, 400 µg/mL, and 600 µg/mL, respectively; EGCG-3Me 200, 400, and 600, epigallocatechin-3-*O*-(3-*O*-methyl)-gallate at 200 µg/mL, 400 µg/mL, and 600 µg/mL, respectively; SB 2, Single Bond 2 (3M ESPE, St. Paul. MN, USA).

Figure 4 presents the CLSM images. Green/red fluorescence indicated live/dead bacteria separately. The green fluorescence overspread the whole vision of SB 2 and showed substantial overlapping of green light spots, which indicated dense biofilms on the specimen. Virtually no red fluorescence occurred with in SB 2. The area of green fluorescence of EGCG/EGCG-3Me decreased with increasing concentration. There was a high proportion of dead bacteria with EGCG 400, EGCG-3Me 400, and EGCG-3Me. Virtually no biofilm formed with EGCG 600/EGCG-3M 600, for which green fluorescence and red fluorescence were rare.

### 3.3. Microtensile Bond Strength

Table 3 shows the mean MTBS values and standard deviation before and after thermocycling. Two-way ANOVA revealed that time had a significant effect on the MTBS (*p =* 0.004), but that the type of adhesive was insignificant (*p =* 0.676). The interaction between time and type of adhesive was insignificant (*p =* 0.142). There were no significant differences between SB 2 and the experimental groups in the immediate bond strength (*p =* 0.971). After thermocycling, the MTBS of the control group reduced significantly (*p =* 0.028), whereas the MTBS of the experimental groups were significantly higher than that of control group (*p* < 0.05). No significant reduction was observed when EGCG/EGCG-3Me was incorporated into the adhesive (*p* > 0.05).

### 3.4. Inhibition of Dentin-Originated Collagen Proteases Activities

**Figure 5:**
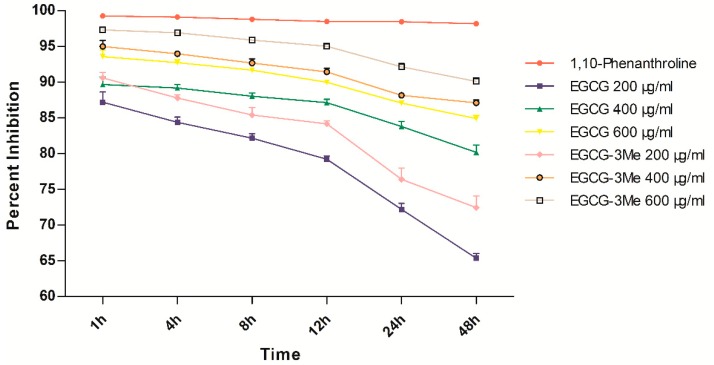
The percent inhibition of dentin-originated collagen proteases activities by increasing concentrations of EGCG and EGCG-3Me at each time points. EGCG, epigallocatechin-3-gallate; EGCG-3Me, epigallocatechin-3-*O*-(3-*O*-methyl)-gallate.

Figure 5 shows the inhibition of dentin-originated collagen proteases activities by increasing concentrations of EGCG/EGCG-3Me at each time point. Two-way ANOVA showed that time (*p* < 0.001) and the experimental solutions (*p* < 0.001) had a significant effect on the inhibition of collagen proteases activities. The interaction between time and the experimental solutions was significant (*p* < 0.001). The positive control inhibited the activities of collagen proteases nearly 100% throughout 48 h. The EGCG 200 concentration inhibited more than 85% collagen proteases activities within 1 h, which was the lowest among all experimental groups. At the same time point, EGCG-3Me 600 inhibited nearly 97% of the collagen proteases activities, which was the highest. The inhibition of dentin proteases activities tended to decrease with time and decreased more rapidly after 12 h among the experimental groups. However, EGCG 600, EGCG-3Me 400, and EGCG-3Me 600 still inhibited more than 90% of the collagen proteases activities after 48 h. The EGCG-3Me concentration showed better inhibition than EGCG at the same concentration, especially after 12 h. In addition, the greater the concentration of EGCG and EGCG-3Me, the greater was the inhibition.

### 3.5. Degree of Conversion

**Figure 6:**
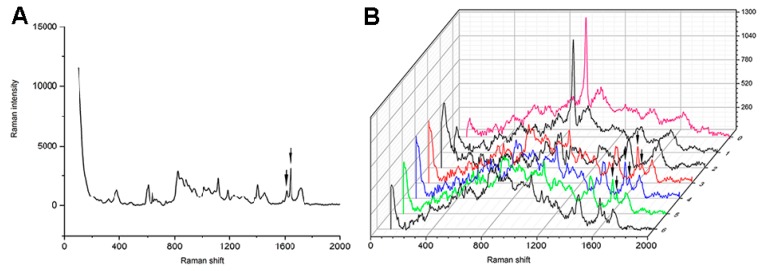
Micro-Raman spectrum of SB 2: The solid arrows represent the adhesive peaks before (**A**) and after (**B**) curing at the Raman shift of 1608 cm^−1^ and at 1640 cm^−1^. The pink Raman spectrum is the representative spectrum of mineralized dentin; the red Raman spectrum is the representative spectrum of the adhesive at the bottom of the HL; the blue Raman spectrum is the representative spectrum of the adhesive at the middle of the HL; and the green Raman spectrum is the representative spectrum of the adhesive at the surface of the HL. HL, hybrid layer.

The mean values and standard deviations of the DC are presented in Table 4. The representative micro-Raman spectrum of SB 2 is shown in Figure 6 and representative micro-Raman spectrum of the experimental adhesives is presented in the Supplementary Material. Two-way ANOVA showed that depth (*p* < 0.001) and adhesives (*p* < 0.001) had a significant effect on the DC of the adhesives. The interaction between depth and adhesives was significant (*p* = 0.049). Depth had no significant effect on the DC of adhesives, for SB 2, EGCG 200, EGCG-3Me 200, and EGCG-3Me 400. For EGCG 400 (*p =* 0.005) and EGCG-3Me 600 (*p* = 0.027), the DC of the adhesives at the bottom of the HL were significantly lower than that at the adhesive–composite interface. For EGCG 600, the DC of adhesives at the bottom of the HL (*p* = 0.004) and the middle of the HL (*p* = 0.016) were significantly lower than that at the adhesive–composite interface. Within the adhesive–composite interface, the DC of the adhesives were significantly decreased in EGCG 600 (*p* < 0.001) and EGCG-3Me 600 (*p* = 0.015), compared to SB 2. The DC was higher for EGCG-3Me 600 than for EGCG 600 (*p* = 0.043). Within the middle of the HL, the DC in EGCG 200 (*p* = 0.205) and EGCG-3Me 200 (*p* = 0.219) were not decreased, compared to SB 2. The DC was higher in EGCG-3Me 600 than in EGCG 600 (*p* < 0.001). Within the bottom of the HL, the DC in EGCG 200 (*p* = 0.536) and EGCG-3Me 200 (*p* = 0.514) was not decreased, compared to SB 2. The DC was higher in EGCG-3Me 600 than in EGCG 600 (*p =* 0.003).

## 4. Discussion

The instability of the resin–dentin interface, which is primarily caused by secondary caries and MMP induced degradation of the HL, is one of the most important causes of resinous restorations failure [5]. Recent studies have focused on developing adhesive systems with an antibacterial effect and preferable bonding stability [34,42,43,44]. The antibacterial effect and bonding durability of EGCG/EGCG-3Me incorporated dental adhesive were evaluated in the present study. EGCG and EGCG-3Me incorporated dental adhesives showed anti-*S. mutans* effect and enhanced bonding stability without harming the DC of the adhesive; however, EGCG-3Me performed better than EGCG. Thus, the hypothesis was accepted.

Most of current adhesives have no antibacterial effect and resinous materials tend to accumulate more bacteria [13]. Researchers have attempted to introduce antibacterial agent into adhesives and have shown promising results [10,21]. EGCG, a flavonoid with several hydroxyl groups, has recently been introduced into the dental field because of several promising benefits such as its antibacterial effect, inhibition of MMP and protection of the teeth from erosion [32,34]. Its *O*-methylated derivative, EGCG-3Me, has also been proven to pose better absorption, stability, bioavailability, lipophilicity, and anti-tubercle bacillus activity than EGCG [38,39,40]. Several indexes were tested in present study to evaluate the antibacterial effect of EGCG/EGCG-3Me incorporated adhesive. The results of OD 600 values indicated that all experimental groups showed significantly antibacterial effect except for EGCG 200 and the antibacterial effect of EGCG-3Me was better than EGCG at the same concentration. SEM observations showed that *S. mutans* biofilm at the surface of the cured adhesive became sparser as the concentration of EGCG/EGCG-3Me increased, which indicated a concentration-dependent antibacterial effect. This finding was similar to the findings by Du et al [34]. Compared to EGCG, EGCG-3Me more strongly inhibited *S. mutans* biofilm formation. For the CLSM test, the amount of bacteria on the surface of the modified adhesive decreased as the concentration of EGCG and EGCG-3Me increased, although the proportion of dead bacteria did not increase significantly. We speculate that the antibacterial mechanism of the cured EGCG and EGCG-3Me modified adhesive is primarily attributable to influence of the initial bacterial adhering to the surface. The antibacterial mechanism of the EGCG- and EGCG-3Me modified adhesives was similar to that of DMAE-CB modified adhesive, which inhibited *S. mutans* on the surface but had no effect on bacteria away from the surface [44]. The antibacterial effect of EGCG and EGCG-3Me was expressed after the polymerization of the adhesive, which was different from the antibacterial effect of the pretreatment solution or the primer of the self-etching adhesive [49,50]. A former study [34] demonstrated that the antibacterial effect of polymerized EGCG modified adhesive would not reduce after one-month water storage. Being limited, the present study did not involve any test about the long-term antibacterial effect. It is of interest to evaluate the long-term antibacterial effect of EGCG and EGCG-3Me modified adhesive in the further study.

Several methods can enhance the bonding durability of adhesives: application of collagen cross-linkers, use of MMP inhibitors, and the ethanol-wet bonding technique [51]. Chlorhexidine is one of the most studied agents due to its anti-proteases effects, while it also has some limitations: Low concentration of CHX would impair the DC and mechanical property of adhesive [52]. Besides, CHX is easy to release from the resin system at a low pH and consequently fail to protect the collagen of the HL [53]. Fortunately, researchers adhere to seek other solutions. Former studies proved that the MTBS of EGCG modified adhesive did not decrease within six months water storage, and dentin pretreatment with EGCG/water solution or EGCG/ethanol solution could improve the bonding stability [34,35]. Inspired by this, we incorporated EGCG and EGCG-3Me into the commercial adhesive SB 2 and tested the bonding durability of the adhesive in our study. Thermocycling is a commonly used thermal fatigue method to evaluate bond durability. It simulates the thermal changes in the oral cavity caused by eating, drinking, and breathing [54]. Three hundred cycles of thermocycling significantly decrease bond strength and 10,000 thermal cycles correspond to one year of clinical function [55,56]. Moreover, thermocycling for 5000 cycles had been proven to be an effective method to accelerate collagen degradation of adhesive treated dentin [57]. Thus, 5000 cycles of thermocycling was used in this study to simulate six months of clinical function. The MTBS of SB 2 decreased significantly (*p* < 0.001) after 5000 cycles of thermocycling, while the MTBS of EGCG and EGCG-3Me showed no significant decrease. When EGCG and EGCG-3Me were each incorporated into the adhesive, the resin–dentin interface could be protected from thermocycling effects resulting in improved bonding stability. This finding indicated that EGCG could be an effective agent with improved bonding performance. Our results were in line with a previous study [34], which demonstrated that the EGCG modified adhesive had better bonding stability after six months of aging in water storage. The researchers have attributed the effect of EGCG on improving the bonding stability to its inhibition to the matrix-bound MMPs. A previous study [58] demonstrated that EGCG might inhibit the activity and expression of osteoblast-derived MMP-2, -9 and -13. However, no reports have shown direct proof that EGCG/EGCG-3Me could inhibit the activities of dentin-originated proteases. To our knowledge, the present study is the first directly demonstrate that EGCG and EGCG-3Me can inhibit the activities of dentin-originated collagen proteases, with the concentration higher than 200 µg/mL. This may explain the maintained MTBS of EGCG and EGCG-3Me modified adhesives after 5000 cycles of thermocycling. The mechanisms of the inhibition of proteases by EGCG and EGCG-3Me are not completely understood. Some researchers suggest that EGCG could inhibit MMP-9 and CT-B by binding to the catalytic site or to an allosteric site of proteases by changing the enzyme conformation or by a zinc-chelating effect [59]. The EGCG-3Me substrate is an *O*-methylated derivative of EGCG and may inhibit the activity of proteases in the same manner. Furthermore, we found that EGCG-3Me had a higher proteases inhibition percentage after 48 h, especially at the concentrations of 400 µg/mL and 600 µg/mL. This indicated that EGCG-3Me had a more enduring proteases inhibition property, compared to EGCG. This observation may be because of the higher stability of EGCG-3Me. Further study should be conducted to determine whether the inhibition of proteases is affected by the addition of a methyl group in the galloyl moieties. This addition is the main difference between the molecular structure of EGCG and EGCG-3Me.

**Figure 7:**
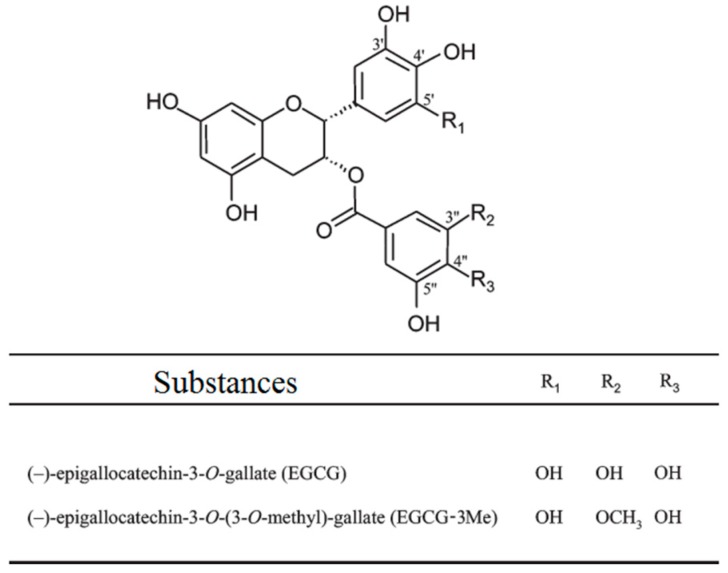
The chemical structure of EGCG and EGCG-3Me. EGCG, epigallocatechin-3-gallate; EGCG-3Me, epigallocatechin-3-*O*-(3-*O*-methyl)-gallate.

Single Bond 2 is a commercial adhesive with a unique formulation, and possible negative effects on bonding efficiency when incorporating other substances into it needs to be considered [60]. Based on a technique described in a previous study [34], we used ethanol as the solvent to dissolve EGCG and EGCG-3Me and then added them separately to the adhesive. This action can minimize the influence of the change in the adhesive formulation. The immediate MTBS test showed that incorporating EGCG or EGCG-3Me did not compromise the bonding property of the adhesive. The DC of the adhesive revealed the polymerization degree of the adhesive and was tightly related to the adhesive property [61]. Therefore, we assessed the effect of incorporating different concentrations of EGCG and EGCG-3Me into SB 2 on the DC of the adhesive. To the best of our knowledge, this is the first study to use a real dentin-bonding model to evaluate the effect of incorporating EGCG and EGCG-3Me on the polymerization of adhesive resin at different depths of the HL. The DC of the modified adhesive was evaluated at three positions: (1) the adhesive–composite interface; (2) the middle of the HL; and (3) the bottom of the HL that adjoined the dentin. Regardless of the influence of the depth of the HL, the DC was not compromised at the 200 µg/mL concentration of EGCG and EGCG-3Me. The DC of EGCG 600 and EGCG-3Me 600 was significantly decreased, compared to SB 2. However, the DC of EGCG-3Me 600 was higher than that of EGCG 600, indicating EGCG-3Me had a relatively smaller effect on the DC, compared to EGCG. With regard to the depth of the HL, our results indicated that etched dentin or resinous restorations would not affect the DC of SB 2 during clinical practice. After being modified with EGCG or EGCG-3Me, the depth of the HL had no influence on the DC at a low concentration. However, the depth of the HL affected the DC when the concentration of EGCG/EGCG-3Me was high (EGCG 400, EGCG 600, and EGCG-3Me 600). At the same concentrations, EGCG affected the DC more substantially, compared to EGCG-3Me. These factors may be attributable to the differences in the chemical structure between EGCG and EGCG-3Me (Figure 7). EGCG has a free radical scavenging effect, which may disturb the free radical polymerization of the adhesive [62], whereas EGCG-3Me has fewer hydroxyl groups than EGCG and thus may have a weaker influence on the polymerization of an adhesive [63]. Incomplete solvent evaporation compromises the strength of resin tags and causes a lack of homogeneity within the infiltrated adhesives [64]. The high concentration of the added substances in our study may have intensified the problem and lowered the DC of the adhesive in the middle of the HL and in the bottom of the HL.

## 5. Conclusions

Within the limitation of this study, EGCG and EGCG-3Me modified adhesives showed effective antibacterial activity against *S. mutans* and enhanced bonding stability to dentin. In addition, the inhibitory effect of EGCG and EGCG-3Me on dentin-originated proteases was demonstrated for the first time with direct evidence. EGCG-3Me had better antibacterial effect, protease inhibition, and less influence on the DC, compared to EGCG. Therefore, adhesive modification by EGCG-3Me at a concentration of 400 µg/mL may be a more promising method to improve the long-term use of resinous restorations. This research may help foster the development of new methods to enhance the long-term use of resinous restorations.

## Figures and Tables

**Table 1:** The adhesive used in this study.

Materials	Composition	Application Procedures
Adper Single Bond 2 (3 M ESPE, St. Paul, MN, USA)	bis-GMA; HEMA; Polyalkenoic; Acid copolymer; Photoinitiators; Ethanol; Water.	Phosphoric acid-etching for 15 s; rinse with water for 10 s; dry with paper points; apply two coats of adhesive; air-dry for 5 s; light cure for 10 s.

bis-GMA, bisphenol-A diglycidyl ether dimethacrylate; HEMA, 2-hydroxyethyl methacrylate.

**Table 2:** The OD 600 values of the adhesives.

Adhesive	OD 600 Values
SB 2	0.438 ± 0.029 ^a,^
EGCG 200	0.379 ± 0.039 ^a,^
EGCG 400	0.292 ± 0.026 ^b^
EGCG 600	0.219 ± 0.037 ^c^
EGCG-3Me 200	0.241 ± 0.037 ^b,c^
EGCG-3Me 400	0.201 ± 0.025 ^c^
EGCG-3Me 600	0.136 ± 0.028 ^d^

The data are presented as the mean ± the standard deviation. ^a,b,c,d^ For each vertical column, values with identical lowercase letters indicate no significant difference (*p* > 0.05). EGCG 200, 400, and 600, epigallocatechin-3-gallate at 200 µg/mL, 400 µg/mL, and 600 µg/mL, respectively; EGCG-3Me 200, 400, and 600, epigallocatechin-3-*O*-(3-*O*-methyl)-gallate at 200 µg/mL, 400 µg/mL, and 600 µg/mL, respectively; OD 600, optical density of 0.5 at 600 nm; SB 2, Single Bond 2 (3M ESPE, St. Paul, MN, USA).

**Table 3:** Microtensile bond strength (MPa).

Time	Adhesive						
	SB 2	EGCG 200	EGCG 400	EGCG 600	EGCG-3Me 200	EGCG-3Me 400	EGCG-3Me 600
Immediate	33.44 ± 6.43 ^a,1^	32.64 ± 5.30 ^a,1^	30.38 ± 3.64 ^a,1^	30.20 ± 4.83 ^a,1^	30.43 ± 5.20 ^a,1^	31.20 ± 4.17 ^a,1^	31.51 ± 3.08 ^a,1^
After Thermocycling	20.80 ± 2.22 ^b,1^	27.09 ± 2.96 ^a,2^	27.61 ± 1.40 ^a,2^	27.76 ± 1.83 ^a,2^	27.82 ± 2.36 ^a,2^	29.60 ± 2.04 ^a,2^	29.99 ± 2.26 ^a,2^

The data are presented as the mean ± the standard deviation. ^a,b,1,2^ For each vertical column, values with identical lowercase letters indicate no significant change before and after the thermocycling process within the same adhesive (*p* > 0.05). For each horizontal row, values with identical numbers indicate no significant difference (*p* > 0.05). EGCG 200, 400, and 600, epigallocatechin-3-gallate at 200 µg/mL, 400 µg/mL, and 600 µg/mL, respectively; EGCG-3Me 200, 400, and 600, epigallocatechin-3-*O*-(3-*O*-methyl)-gallate at 200 µg/mL, 400 µg/mL, and 600 µg/mL, respectively; SB 2, Single Bond 2.

**Table 4:** Degree of conversion values (%).

Adhesive	Degree of Conversion (%)		
	Adhesive-Composite Interface	Middle of the Hybrid Layer	Bottom of the Hybrid Layer that Adjoined the Dentin
**SB 2**	73.14 ± 0.85 ^a,1^	72.23 ± 0.89 ^a,1^	72.00 ± 1.84 ^a,1^
**EGCG 200**	71.05 ± 1.49 ^a,c,1^	69.05 ± 0.93 ^a,c,1^	68.90 ± 0.38 ^a,d,1^
**EGCG 400**	69.34 ± 2.02 ^a,c,1^	65.53 ± 0.45 ^c,1,2^	62.56 ± 1.85 ^c,2^
**EGCG 600**	62.49 ± 2.61 ^b,1^	56.03 ± 1.11 ^b,2^	53.83 ± 1.89 ^b,2^
**EGCG-3Me 200**	71.26 ± 1.14 ^a,c,1^	69.11 ± 2.84 ^a,c,1^	68.84 ± 2.66 ^a,d,1^
**EGCG-3Me 400**	69.55 ± 1.66 ^a,c,1^	67.47 ± 1.16 ^c,1^	65.91 ± 2.80 ^c,d,1^
**EGCG-3Me 600**	67.42 ± 1.70 ^c,1^	65.23 ± 1.84 ^c,1,2^	62.03 ± 1.95 ^c,2^

^a,b,c,d,1,2^ For each vertical column, values with identical lowercase letters indicate no significant difference (*p* > 0.05). For each horizontal row, values with identical numbers indicate no significant difference between the different depths of the hybrid layer within the same adhesive system (*p* > 0.05). EGCG 200, 400, and 600, epigallocatechin-3-gallate at 200 µg/mL, 400 µg/mL, and 600 µg/mL, respectively; EGCG-3Me 200, 400, and 600, epigallocatechin-3-*O*-(3-*O*-methyl)-gallate at 200 µg/mL, 400 µg/mL, and 600 µg/mL, respectively; SB 2, Single Bond 2 (3M ESPE, St. Paul, MN, USA).

## Supplementary Materials

The following are available online at www.mdpi.com/1996-1944/10/2/183/s1.
