# Supplementary material for: Epigallocatechin-3-gallate and Epigallocatechin-3-O-(3-O-methyl)-gallate Enhance the Bonding Stability of an Etch-and-Rinse Adhesive to Dentin
Source: Materials (Basel). 2017 Feb 15;10(2):183. doi: 10.3390/ma10020183 (PMC5459131; doi:10.3390/ma10020183)
Supplement: Supplementary file 1 [file materials-10-00183-s001.zip › Supplementary material/Figure legends S1.docx]

[1] Supl. 1. Micro-Raman spectrum of EGCG 200: The solid arrows represent the adhesive peaks before (**A**) and after (**B**) curing at the Raman shift of 1608 cm^−1^ and at 1640 cm^−1^. The pink Raman spectrum is the representative spectrum of mineralized dentin; the red Raman spectrum is the representative spectrum of the adhesive at the bottom of the HL; the blue Raman spectrum is the representative spectrum of the adhesive at the middle of the HL; and the green Raman spectrum is the representative spectrum of the adhesive at the surface of the HL. HL, hybrid layer.

[2] Supl. 2. Micro-Raman spectrum of EGCG 400: The solid arrows represent the adhesive peaks before (**A**) and after (**B**) curing at the Raman shift of 1608 cm^−1^ and at 1640 cm^−1^. The pink Raman spectrum is the representative spectrum of mineralized dentin; the red Raman spectrum is the representative spectrum of the adhesive at the bottom of the HL; the blue Raman spectrum is the representative spectrum of the adhesive at the middle of the HL; and the green Raman spectrum is the representative spectrum of the adhesive at the surface of the HL. HL, hybrid layer.

[3] Supl. 3. Micro-Raman spectrum of EGCG 600: The solid arrows represent the adhesive peaks before (**A**) and after (**B**) curing at the Raman shift of 1608 cm^−1^ and at 1640 cm^−1^. The pink Raman spectrum is the representative spectrum of mineralized dentin; the red Raman spectrum is the representative spectrum of the adhesive at the bottom of the HL; the blue Raman spectrum is the representative spectrum of the adhesive at the middle of the HL; and the green Raman spectrum is the representative spectrum of the adhesive at the surface of the HL. HL, hybrid layer.

[4] Supl. 4. Micro-Raman spectrum of EGCG-3Me 200: The solid arrows represent the adhesive peaks before (**A**) and after (**B**) curing at the Raman shift of 1608 cm^−1^ and at 1640 cm^−1^. The pink Raman spectrum is the representative spectrum of mineralized dentin; the red Raman spectrum is the representative spectrum of the adhesive at the bottom of the HL; the blue Raman spectrum is the representative spectrum of the adhesive at the middle of the HL; and the green Raman spectrum is the representative spectrum of the adhesive at the surface of the HL. HL, hybrid layer.

[5] Supl. 5. Micro-Raman spectrum of EGCG-3Me 400: The solid arrows represent the adhesive peaks before (**A**) and after (**B**) curing at the Raman shift of 1608 cm^−1^ and at 1640 cm^−1^. The pink Raman spectrum is the representative spectrum of mineralized dentin; the red Raman spectrum is the representative spectrum of the adhesive at the bottom of the HL; the blue Raman spectrum is the representative spectrum of the adhesive at the middle of the HL; and the green Raman spectrum is the representative spectrum of the adhesive at the surface of the HL. HL, hybrid layer.

[6] Supl. 6. Micro-Raman spectrum of EGCG-3Me 600: The solid arrows represent the adhesive peaks before (**A**) and after (**B**) curing at the Raman shift of 1608 cm^−1^ and at 1640 cm^−1^. The pink Raman spectrum is the representative spectrum of mineralized dentin; the red Raman spectrum is the representative spectrum of the adhesive at the bottom of the HL; the blue Raman spectrum is the representative spectrum of the adhesive at the middle of the HL; and the green Raman spectrum is the representative spectrum of the adhesive at the surface of the HL. HL, hybrid layer.
