# Supplementary figures and images for: Epigallocatechin-3-gallate and Epigallocatechin-3-O-(3-O-methyl)-gallate Enhance the Bonding Stability of an Etch-and-Rinse Adhesive to Dentin
Source: Materials (Basel). 2017 Feb 15;10(2):183. doi: 10.3390/ma10020183 (PMC5459131; doi:10.3390/ma10020183)

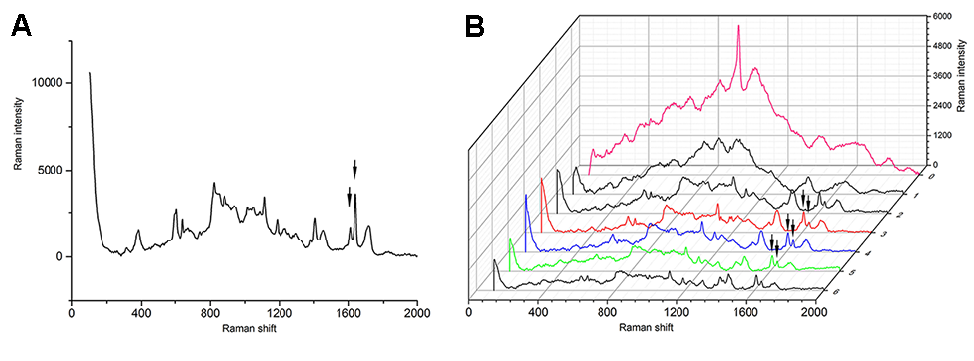

Supplement: Supplementary file 1 [file materials-10-00183-s001.zip › Supplementary material/Figure S1.tif]

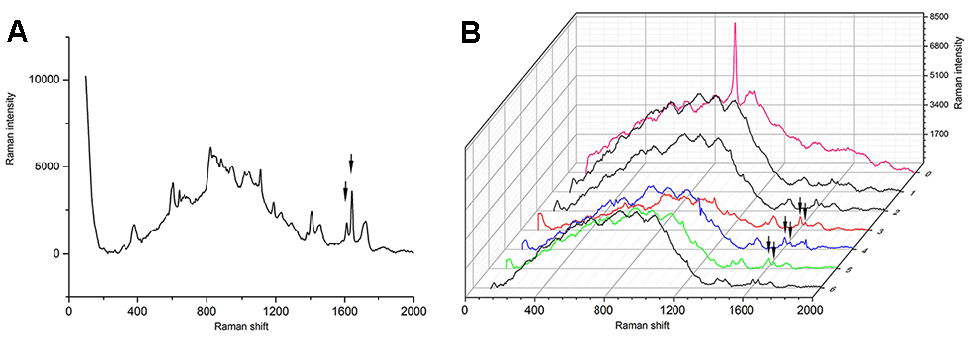

Supplement: Supplementary file 1 [file materials-10-00183-s001.zip › Supplementary material/Figure S2.tif]

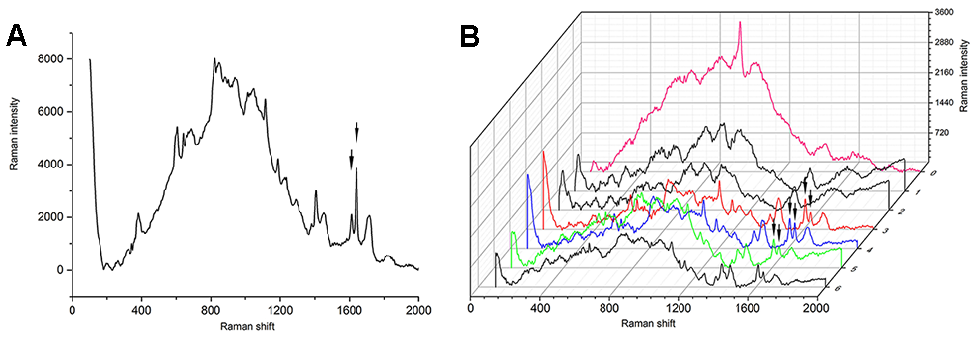

Supplement: Supplementary file 1 [file materials-10-00183-s001.zip › Supplementary material/Figure S3.tif]

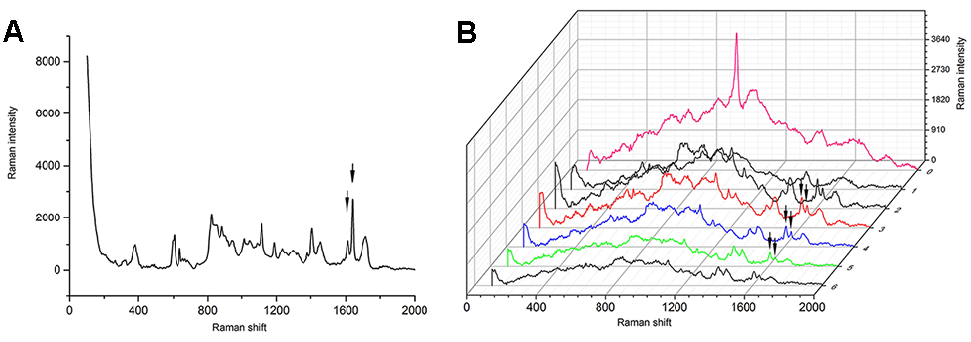

Supplement: Supplementary file 1 [file materials-10-00183-s001.zip › Supplementary material/Figure S4.tif]

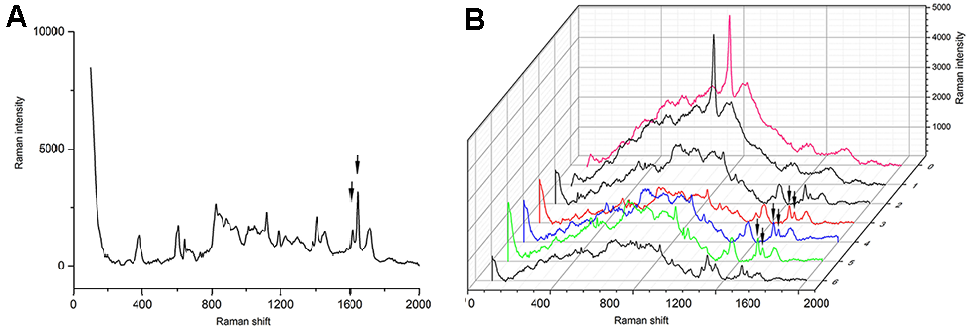

Supplement: Supplementary file 1 [file materials-10-00183-s001.zip › Supplementary material/Figure S5.tif]

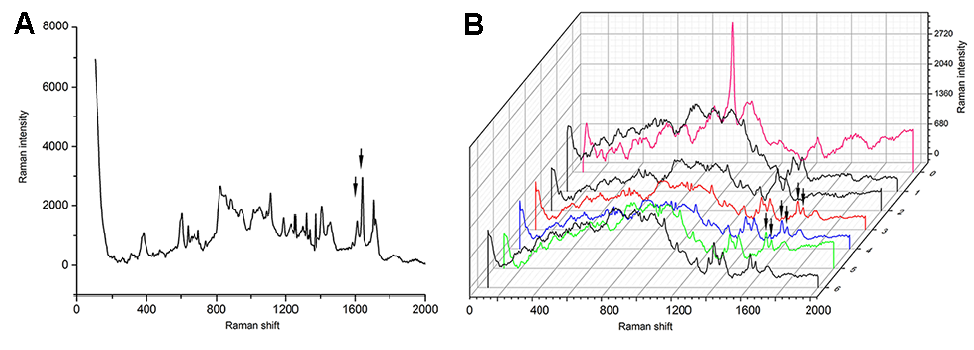

Supplement: Supplementary file 1 [file materials-10-00183-s001.zip › Supplementary material/Figure S6.tif]
